# Poster Session I - A91 SECONDARY BILE ACIDS MEDIATE METABOLIC RECOVERY POST-BARIATRIC SURGERY

**DOI:** 10.1093/jcag/gwaf042.091

**Published:** 2026-02-13

**Authors:** N Nathan, J Yadav, K J Schwenger, Y Ghorbani, B K Tsankov, J P Allard, D J Philpott

**Affiliations:** Immunology, University of Toronto Temerty Faculty of Medicine, Toronto, ON, Canada; Immunology, University of Toronto Temerty Faculty of Medicine, Toronto, ON, Canada; Immunology, University of Toronto Temerty Faculty of Medicine, Toronto, ON, Canada; University Health Network, Toronto, ON, Canada; Immunology, University of Toronto Temerty Faculty of Medicine, Toronto, ON, Canada; University Health Network, Toronto, ON, Canada; Immunology, University of Toronto Temerty Faculty of Medicine, Toronto, ON, Canada

## Abstract

**Background:**

Type 2 diabetes (T2D) is a growing problem in the developed world. Today, 1 in 10 Canadian adults live with diagnosed T2D and is currently the 8^th^ leading cause of global mortality, taking the lives of close to 2 million people annually. With T2D incidences rising annually and associated with multiple comorbidities, there is an urgent need for novel T2D interventions. While T2D can be treated with pharmacologic intervention or lifestyle changes, weight-loss surgeries, such as bariatric surgery (BS), have been shown to promote long-term diabetes remission, often outperforming the efficacy of other interventions. Although the mechanistic nature of metabolic improvement post-BS is not well characterized, our group has revealed a causal role for the gut microbiome in improving host metabolism post-surgery. Specifically, we transferred gut microbes from patients’ feces before and following BS into germ-free (GF), Western diet-fed (WD) mice. Indeed, mice receiving a fecal microbiota transplant (FMT) from patients’ post-BS showed increased insulin sensitivity and normalized blood glucose levels compared to animals colonized with fecal samples from the same patient before BS, independent of weight.

**Aims:**

We hypothesize that the microbiome post-BS improves host metabolism indirectly by modulating systemic inflammation.

Aim 1: Identification of metabolic signatures associated with metabolic improvements post-BS.

Aim 2: Validation of metabolomic targets in vivo.

Aim 3: In vitro mechanistic validation of putative targets.

**Methods:**

Metabolomics was used to determine microbiota-derived metabolites associated with metabolic improvements post-BS. Gnotobiotic mice were generated by FMT with patient fecal samples. Mice were then fed a Western-diet for 16 weeks and sacrificed. Tissues were collected for mRNA analyses. Tissues were also fixed and stained for gross pathology.

Mechanistic studies were performed *in vivo* by feeding mice a chow diet supplemented with metabolites of interest for 8 weeks. Mice were sacrificed in the same manner as gnotobiotic mice.

*In vitro* studies were performed using 3T3 adipocytes and T84 epithelial cells treated with metabolites.

**Results:**

Aim 1 - We identified deoxycholic acid (DCA) as being significantly reduced in patient feces post surgery. Concomitantly, the reduction in DCA was transferred to mice following FMT, suggesting that this alteration is microbially mediated.

Aim 2 - DCA impaired glucose handling in mice in a weight-independent manner.

Aim 3 - *In vitro*, DCA induced inflammation and cellular stress. DCA impaired barrier function in polarized intestinal monolayers. In adipocytes, DCA limited adipogenesis and increased pro-inflammatory gene expression.

**Conclusions:**

In sum, this data highlights a critical role for DCA and secondary bile acid metabolism in the modulation of host metabolism.

**Funding Agencies:**

CIHR

